# Lifecourse immunization and adaptation to changing needs of India’s universal immunization program: theory to practice

**DOI:** 10.3389/fpubh.2026.1938980

**Published:** 2026-10-28

**Authors:** Pritu Dhalaria, Pawan Kumar, Sanjay Kapur, Pretty Priyadarshini, Ajeet Kumar Singh, Paras Jain, Bhupendra Tripathi, Arindam Ray

**Affiliations:** 1 Immunization Technical Support Unit, Ministry of Health and Family Welfare, Government of India, New Delhi, India; 2 Immunization Division, Ministry of Health and Family Welfare, Government of India, New Delhi, India; 3 John Snow India, New Delhi, India; 4 Gates Foundation, New Delhi, India

**Keywords:** ageing, health systems, life course approach, life course immunization, life expectancy, vaccine preventable diseases

## Abstract

The Expanded Program on Immunization, introduced in 1974, marks a pivotal milestone in public health and celebrates its 50th anniversary in 2024. Originally targeting childhood immunization, the program has expanded to include adolescents and adults, driven by the evolving epidemiology of vaccine-preventable diseases and age-related immunosenescence. The COVID-19 pandemic highlighted the vulnerabilities of older populations, reinforcing the need for a lifecourse approach (LCA) to immunization. The article explores the integration of LCA into global health systems, focusing on low-and middle-income countries (LMICs) and India. The article discusses the critical factors and policy-level efforts that India and countries with similar contexts need to consider in expanding immunization programs across the lifecourse.

## 1 Introduction

The global eradication of smallpox in 1980, following a landmark call to action by the World Health Assembly, remains a remarkable achievement. Building on this momentum, the 1974 launch of the “Expanded Program on Immunization” (EPI) galvanized nations to establish national immunization programs (1). This initiative adopted a “*pediatric approach*,” focusing primarily on immunizing children and pregnant women to reduce childhood morbidity and mortality. Gradually, the breadth of protection (BOP) against Vaccine Preventable Diseases (VPDs) was extended beyond childhood to adolescents and adults for certain diseases across the countries. Marking its 50th anniversary in 2024, the EPI represents a transformative milestone in global public health (2). There have been massive improvements in childhood health and vaccination coverage indicators; however, there is a need to expand efforts beyond childhood and ensure the benefits of immunization are provided across the life course—a *life course approach* (LCA). Vaccines were introduced to adolescents, adults, and older adults cohorts in different regions and countries at different times; however, the number and reach of such vaccines vary significantly across regions, and coverage remains sub-optimal, primarily in low-and middle-income countries (LMICs) (3).

### 1.1 Global demographic shift

The global population is ageing at an unprecedented rate. According to the United Nations (UN) Decade of Healthy Ageing Report 2021–30, by 2030, adults over 60 will outnumber adolescents and young adults, and one out of every six people is estimated to be 60 or older (4). The population of older adults is projected to grow by 34% by 2030 and double to 2.1 billion by 2050 (5). This demographic shift poses challenges for public health systems in promoting healthy ageing. Global life expectancy has increased from 66.0 in 2000 to 72.6 in 2019 and is expected to rise to 77.1 by 2050 (6). This shift is accompanied by a rising prevalence of non-communicable diseases (NCDs). While NCDs affect individuals across all age groups, their burden is disproportionately higher among older adults due to the accumulation of risk factors, and they become more susceptible to VPDs, disease severity, and complications (7). Ageing is associated with immunosenescence—a gradual functional decline of the immune system that makes older adults more susceptible to infectious diseases. The Immunization Agenda 2030 (IA 2030) also envisions universal access to vaccines across the life course to combat VPDs and strengthen health systems (8).

The rising burden of VPDs highlights the need to expand vaccination programs across the life course to benefit vulnerable populations and alleviate strain on healthcare systems. Global data show that adult immunization significantly reduces the incidence of VPDs and associated healthcare costs (9). Older adults are particularly susceptible to diseases such as influenza, pneumococcal pneumonia, and shingles due to the natural decline of the immune system with age. Vaccination in this age group is vital to reduce morbidity and mortality. Coverage rates vary across WHO regions and are generally higher in high-income countries due to a more robust healthcare infrastructure (10).

The life course approach is a multidisciplinary model that emphasizes dynamic interactions of biological, social, and environmental factors throughout one’s lifetime, influencing health outcomes (11). This approach holds importance given the demographic shift toward increased longevity and persistent social inequalities in health, emphasizing vaccination at every stage—from birth to old age to ensure individuals receive timely immunizations to protect against age-specific diseases. It recognizes that immune responses to vaccines vary by age and several other factors; hence, vaccinating individuals at appropriate ages provides optimal protection against VPDs. It is more important than ever to consider declining immunity in these populations to optimize vaccine effectiveness (12). However, adult vaccination uptake remains suboptimal among at-risk populations, and the life course approach to immunization is yet to be adopted in many countries, as most national immunization programs remain primarily focused on the pediatric population (3).

### 1.2 Disease profile and epidemiological aspects across the life course

The evolving health risks and disease burden across different life stages—children, adolescents, adults, and the older adults present unique challenges and opportunities for vaccination. In 2022, approximately 18 million children worldwide missed the first dose of the DPT (diphtheria, pertussis, and tetanus) vaccine, leaving them vulnerable to outbreaks and jeopardizing herd immunity. These gaps risk individual health and threaten population-level disease control in regions where VPDs have been contained to a large extent (13). Childhood vaccines such as DPT, with >90% efficacy, have significantly reduced global childhood mortality. Similarly, the measles vaccine, with two recommended doses, provides 97% protection, which is critical in outbreak prevention (14). However, diseases like meningococcal meningitis remain a significant threat to adolescents in sub-Saharan Africa, with “high-risk” in the “*meningitis belt*,” with large-scale frequent outbreaks every 5–12 years. Despite the evident need, immunization coverage among adolescents lags behind that of children, largely due to inadequate structured programs and insufficient awareness (15).

While childhood immunization programs have achieved considerable progress, the prevalence of communicable and non-communicable diseases remains a concern among adults and the older adults. The older adults population is disproportionately affected by VPDs such as influenza, pneumococcal disease, and herpes zoster. Influenza alone accounts for up to 72,000 deaths annually among older adults in Europe (16). Pneumococcal disease remains a leading cause of severe pneumonia, sepsis, and meningitis among older populations, particularly in regions with high rates of chronic respiratory illnesses. Although introducing pneumococcal vaccines into adult vaccination schedules has significantly reduced disease incidence, global coverage remains inadequate (17). Herpes zoster (Shingles) affects approximately one in three individuals during their lifetime and presents a significant health risk, particularly for older adults. Vaccination has been shown to effectively lower both the occurrence and the severity of this condition, offering critical protection to this vulnerable population (18). Globally, the integration of life course immunization (LCI) strategies varies widely. In high-income countries (HICs) like the United States, the Centers for Disease Control and Prevention (CDC) recommends vaccinations from infancy through older adulthood, including HPV for adolescents and pneumococcal vaccines for the older adults (19). Similarly, the UK’s National Health Service (NHS) emphasizes flu vaccines for children and older adults, while Japan has included pneumococcal vaccines for the older adults in its national immunization program. Australia’s comprehensive schedule incorporates vaccines like herpes zoster for older adults and HPV for adolescents (20). Hepatitis B affects over 296 million people globally, causing 820,000 deaths annually, primarily from liver disease and cancer. Hepatitis B vaccination is especially critical for high-risk groups, such as healthcare workers and individuals with chronic liver conditions (21, 22).

In contrast, many developing countries remain focused on childhood immunization, with limited vaccine coverage for adolescents and adults. However, countries like Brazil and Mexico are bridging this gap by expanding their immunization programs to include these populations, signaling a growing recognition of LCI’s importance (23, 24). These examples illustrate the varying levels of integration worldwide and emphasize the pressing need for a global framework that addresses immunization across all age groups.

### 1.3 Global landscape of LCI

The global landscape of LCI presents a complex picture of progress and challenges. In many high-income countries, comprehensive vaccination programs cover all stages of life. The United States, the United Kingdom, Japan, and Australia have integrated LCI into their public health strategies, offering vaccines for adolescents (HPV vaccine) and older adults (pneumococcal and shingles vaccines) as part of national schedules (23, 24). These nations serve as models of the life course approach, demonstrating its potential to protect populations across age groups. However, even in these countries, vaccination coverage among adults and the older adults often falls short due to inadequate public awareness, vaccine hesitancy, and logistical challenges.

Conversely, LMICs focus primarily on infant and child immunization to reduce mortality rates. While countries like Brazil and Mexico have expanded their programs to include adolescents and adults, recognizing the burden of diseases like HPV, influenza, and pneumococcal infections, many nations, particularly in Africa and Southeast Asia, still lack widespread vaccine coverage for older age groups. Resource limitations, competing health priorities, and infrastructural challenges hinder the adoption of a comprehensive LCI approach. As of 2021, 65% of WHO member states (128 countries) included influenza vaccination for older adults and high-risk populations, such as healthcare workers and pregnant women. However, only 25% (48 countries) adopted the pneumococcal polysaccharide vaccine for older adults, and in many of these, its use was restricted to high-risk groups (24).

The COVID-19 pandemic reinforced the importance of LCI as countries adapted to vaccinate not only the older adults but also younger adults and adolescents. This global crisis exposed critical weaknesses in vaccine infrastructure, particularly in LMICs, and highlighted the need for resilient health systems capable of delivering vaccines across all age groups (3). This was a stark reminder that a reactive immunization approach is insufficient to address emerging public health threats, and bridging these gaps requires a multifaceted strategy. Limited public awareness and knowledge about immunization among adults and older individuals remain significant challenges, contributing to suboptimal vaccine coverage in these age groups. This lack of understanding often leads to missed opportunities for protection against VPDs, increasing the risk of severe illness and complications. Furthermore, the prevalence of misinformation and misconceptions about vaccines exacerbates hesitancy, undermining efforts to build trust in immunization programs.

Recognizing that immunization is not a one-time event but a continuous process, a life course approach aims to protect individuals against VPDs throughout their lifespans. WHO has recommended a few principles—equity, access, inclusivity, and sustainability (15, 25) to guide the development of policies and systems that target all population segments, focusing more on vulnerable groups such as healthcare workers, pregnant women, and older adults. Sustainability is a critical parameter of LCA, ensuring that immunization efforts are ongoing and adaptable to changing population needs. Integrating LCA into national health policies allows for continuous evaluation and refinement of immunization strategies, enabling countries to address emerging health challenges effectively. The application of LCA is not limited to immunization program design but extends to informing vaccine development. Addressing each age group’s unique health needs ensures that immunization strategies remain relevant and effective across diverse populations.

Examples of successful LCA implementation worldwide highlight its transformative potential. Australia and Japan have integrated LCA into their health systems to ensure vaccines are available to all age groups. These nations have established electronic immunization registries, launched robust public health campaigns, and demonstrated strong political and financial commitments to sustaining immunization programs. Recognizing the importance of continued protection across life stages, both countries have included adolescent and adult vaccines in their national schedules, addressing the evolving demographic landscape and the growing need for comprehensive immunization strategies.

### 1.4 Status of LCI in India

With the world’s largest birth cohort and largest population share, India has made notable progress in immunization. The nation has successfully expanded its immunization programs, attaining significantly high coverage rates for many childhood vaccines. However, a pressing need remains to adopt a life course immunization approach to address the health needs of adolescents, adults, and older adults. This shift is vital to tackling the evolving disease burden, driven by demographic changes, an ageing population, and rising life expectancy, projected to reach 77.0 by 2050 (6). A comprehensive strategy would support healthy ageing and enhance public health outcomes.

## 2 Objective and significance

This narrative review article explores the importance of adopting a life course approach to immunization and its transformative potential in reducing the burden of VPDs. The article examines how countries globally have incorporated LCA into their immunization strategies, provides a comprehensive status update on life course immunization, identifies achievements and gaps, and evaluates factors influencing its successful implementation. Through this lens, the article emphasizes the significance of LCA in addressing challenges across the life span—from childhood and adolescence to adulthood and older age, and highlights its role in enhancing public health, particularly in LMICs. The literature for this narrative review was identified from published peer-reviewed literature as well as relevant government, program and policy documents. The authors used major bibliographic databases and search engines to identify literature relevant to the manuscript’s principal themes, including life-course immunization, adult and adolescent vaccination, immunization program, VPDs, demographic and epidemiological transition, health-system delivery, financing, and implementation.

India’s Universal Immunization Program (UIP) is the world’s largest immunization program, and there is a growing need to expand its focus beyond childhood vaccinations. With its rapidly shifting demographics and increasing proportion of adults and the older adults, the country faces the dual challenges of waning immunity and heightened vulnerability of immunosenescence. This highlights the necessity and scope of integrating LCI into India’s immunization framework, aligning with relevant health policies, and establishing robust systems and strategies to address immunity gaps across all life stages. This paper reinforces LCI’s role in managing emerging zoonotic diseases, addressing the impacts of climate change, and improving resilience against public health emergencies. It goes beyond the conventional focus on childhood VPDs to explore other critical factors, including gender, the One Health concept, antimicrobial resistance (AMR), and socio-economic vulnerability, offering a holistic view of LCI.

The review article evaluates how LCI can reduce the VPD burden while promoting healthy ageing in India’s context—transferable and applicable to other LMICs and regions with similar challenges. It examines systemic and policy-level considerations required to transition to LCI, assessing factors related to vaccine accessibility, affordability, awareness, and the integration of new vaccines into the national immunization program, focusing on key challenges and offering insights into how India can strengthen its immunization policies to meet the evolving health needs of its population.

### 2.1 Region-specific status across age cohorts

Policies and recommendations at regional and global levels have evolved, and countries have introduced vaccines for different age cohorts; however, there has been a visible difference between LMICs and HICs. Global and regional policies, recommendations, and global institutions considerably influence a country’s immunization policies and life course strategies (26). WHO’s recommendations have been a key driving force for many countries to introduce national immunization policies. However, despite recommendations, adult immunization programs have not received sufficient attention, particularly in LMICs.

The WHO categorizes the life course into seven stages—pregnancy, newborn, infant, child, adolescent, adult, and older person (25). However, this article emphasizes an enhanced framework by introducing an additional stage, the “second year of life,” after infancy (Figure 1). This new stage is added to emphasize the unique vulnerabilities and critical health needs of children under five, particularly between the first and second year, who face heightened risks of VPDs due to their developing immune systems. Progress can be observed with a considerable difference across six WHO regions—South East Asia Region (SEAR), European Region (EUR), the Region of the Americas (AMR), the African Region (AFR), the Western Pacific Region (WPR), and the Eastern Mediterranean Region (EMR). WHO mapped the six regions against the seven life course stages for 194 countries using 2019 Joint Reporting Form (JRF) data, jointly done by WHO and UNICEF. At the regional level, there has been considerable progress in the number of countries with universal vaccinations across the life course. Most of the Americas and European regions have vaccines for all age groups in their national immunization programs. Over 50% of countries in the Americas region have included adults, and more than 75% of countries have immunization for older persons and incorporated LCA into their health policies. Only 50% of countries, however, have vaccine recommendations for adolescents. Despite these recommendations, there are gaps in immunization coverage, and the countries in the Americas region still report missed opportunities, particularly among adolescents and adults (25, 27). European countries have made significant progress across life course stages, with approximately 80% have immunization for adolescents and older persons, and nearly 50% of countries have included vaccines for adults.

**Figure 1:**
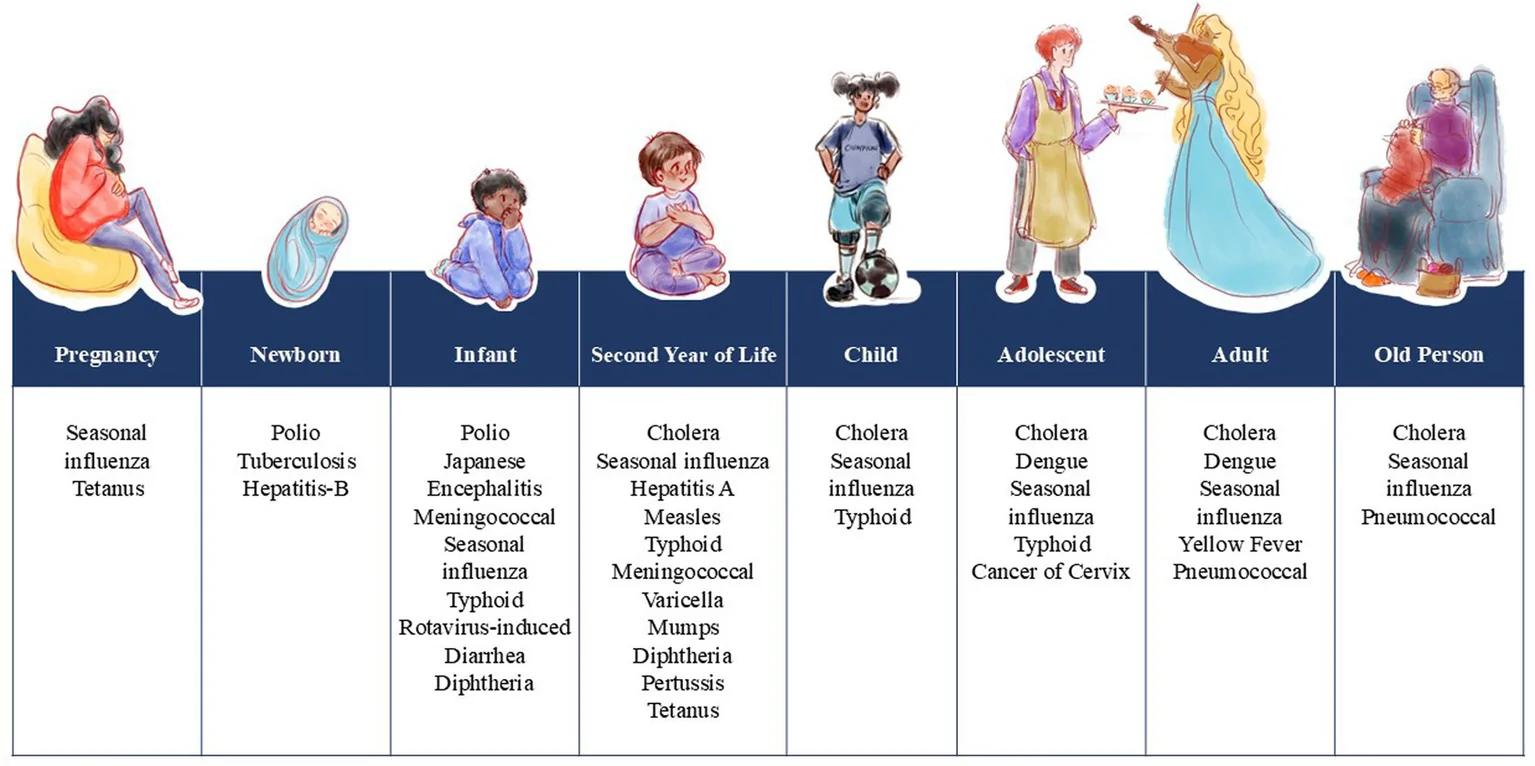
Life course stages (27). The WHO divides the life course into seven stages: pregnancy, newborn, infant, child, adolescent, adult, and older person. This article proposes an enhanced framework that explicitly includes the ‘*second year of life*,’ following infancy. This addition highlights the unique vulnerabilities and critical health needs of children under five, especially between one and 2 years old, who are at an increased risk of VPDs. Every stage of life vividly illustrates the vital message that vaccines are crucial for disease prevention, thereby enhancing the quality of life. The VPDs prevalent at each life stage have also been mentioned. The figure was hand-drawn in Procreate.

Most African countries have incorporated vaccination for newborns and infants; however, only about 70% of these have incorporated vaccines for children over 1 year. Only about a quarter of African countries have vaccine recommendations for adolescents, and about 70% of countries have included vaccines for adults. More than 60% of countries in the Eastern Mediterranean region are remaining to introduce vaccines for adolescents, adults, and older persons. About a quarter of countries in SEAR have vaccinations for adolescents, and about one-third for adults, highlighting the progress and furthering the need for countries to incorporate vaccines for adolescents, adults, and the older adults. The situation in Western Pacific regions is not encouraging for adolescents and adults, although almost half of the countries in the WPR region have covered older persons with certain vaccines. These regional variations accentuate the need to expand vaccination programs beyond childhood.

### 2.2 Global VPD burden and vaccine availability

WHO monitors 12 critical VPDs: Congenital Rubella Syndrome (CRS), Diphtheria, Invasive Meningococcal Disease, Japanese Encephalitis, Measles, Mumps, Pertussis, Poliomyelitis, Rubella, Tetanus (including neonatal tetanus), Typhoid, and Yellow Fever. In 2025, countries reported 5.30 million cases of these diseases to WHO, mainly through the WHO/UNICEF Joint Reporting Form on Immunization (JRF), with India accounting for approximately 11% (0.60 million) (28). Typhoid made up most of these cases, both globally (81%) and in India (95%). Refer to Supplementary Table S1 for year-wise reported cases from 2019 to 2025. India accounted for the highest share of Japanese encephalitis (67%), followed by typhoid (13%), diphtheria (11%), and rubella and CRS (10% each) (Figure 2). Despite the scale of the issue, the JRF data do not differentiate cases by age, limiting insights into how VPDs affect various demographics. The lack of age-specific data, particularly for adult VPD incidence, hinders efforts to develop targeted interventions and accurately assess disease burdens (29). The economic impact is equally pressing. Analysis by the National Sample Survey Organization (NSSO) reveals that over 7% of India’s population is impoverished annually due to healthcare expenses (30). VPDs significantly drive this burden, contributing to widespread morbidity, mortality, and financial strain. The consequences extend beyond individual health, as unvaccinated adults can transmit diseases like pertussis to vulnerable groups, including unimmunized infants (31).

**Figure 2:**
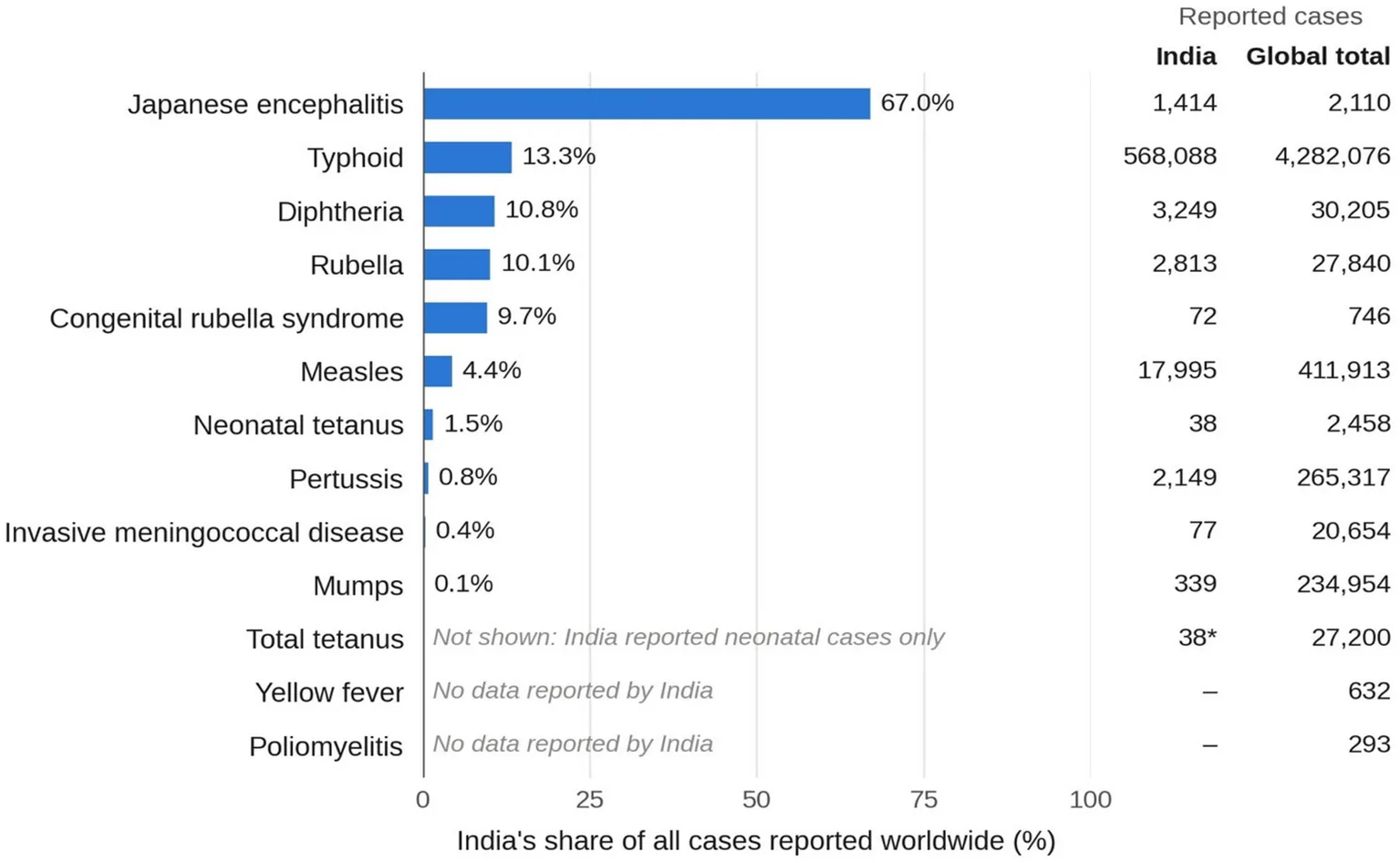
JRF data of VPD cases globally and in India (2025). Bars show the cases India reported for each disease as a percentage of all cases reported to WHO worldwide; the global total includes India. Columns give the underlying counts. Source: WHO Immunization Data Portal, reported cases collected through the WHO/UNICEF Joint Reporting Form on Immunization (poliomyelitis: official country reporting to WHO), accessed 29 September 2026. India reported no data for poliomyelitis or yellow fever. The figure was created using Python (Matplotlib 3.10).

WHO classifies vaccines into two categories—*available vaccines*, which include established immunization for existing diseases, and *pipeline vaccines*, which are in development and target priority diseases for LMICs (Table 1). The pipeline comprises promising candidates for diseases such as Chikungunya, HIV-1, and Shigella. Additionally, WHO supports initiatives to enhance vaccine manufacturing, distribution, and research, particularly focusing on antimicrobial resistance (AMR) and monoclonal antibodies, and developing vaccines for enteric diseases to improve global health equity (28).

**Table 1:** WHO classified vaccines (28).

Category	Diseases/Pathogens
Available vaccines	Cholera, COVID-19 (coronavirus), Dengue, Diphtheria, Hepatitis A and B, *Haemophilus influenzae* type b (Hib), Human papillomavirus (HPV), Influenza, Japanese encephalitis, Malaria, Measles, Meningococcal meningitis, Mumps, Pertussis, Pneumococcal disease, Poliomyelitis, Rabies, Rotavirus, Rubella, Tetanus, Tick-borne encephalitis, Tuberculosis, Typhoid, Varicella, Yellow Fever
Pipeline vaccines	Chikungunya, Enterotoxigenic *Escherichia coli*, Group A Streptococcus (GAS), Group B Streptococcus (GBS), Herpes Simplex Virus, HIV-1, Improved Influenza Vaccines, Malaria, *Neisseria gonorrhoeae*, Nontyphoidal Salmonella Disease, Norovirus, Paratyphoid fever, Respiratory Syncytial Virus (RSV), Schistosomiasis Disease, Shigella, Tuberculosis

### 2.3 Vaccine availability in India

Table 2 highlights vaccine availability through UIP and through the private sector. While essential vaccines like Diphtheria, Hepatitis B, Measles, and Tetanus are part of the UIP for children under age five, pregnant women, and adolescent girls, some key vaccines, such as Hepatitis A, Influenza, and typhoid conjugate vaccine (TCV), remain limited to private healthcare settings. The table also shows the critical gap in adult and older adults immunization, such as pneumococcal disease and Japanese Encephalitis, primarily targeted at children, leaving older age groups under-protected.

**Table 2:** Vaccine availability—global and India (36, 74, 88–91).

Age cohort	WHO	India	
		UIP schedule	Private vaccination schedule
Pregnant women	Routine vaccines: COVID-19, Hepatitis A*, Hepatitis B, Influenza (Inactivated), Mpox***, Meningococcal (ACWY)^, Meningococcal (B)**, Polio (IPV)***, Respiratory syncytial virus (RSV), Tdap. Non-routine: Anthrax#, Cholera**, Dengue**, Ebola**, Japanese Encephalitis**, Tick-borne Encephalitis^, Vi polysaccharide##, Yellow Fever**	Routine vaccines: Td. Non-routine vaccines: COVID-19, Yellow Fever** (It should be given only under supervision by the infectious disease specialist).	Routine vaccines: Td or Tdap, COVID-19, Polio (IPV)***, Influenza (Inactivated), Hepatitis A*, Hepatitis B, Meningococcal (ACWY)^, Meningococcal (B)**
Newborn	Hepatitis B, BCG, OPV, RSV	BCG, OPV, Hep B	BCG, OPV, Hep B, RSV
Infant	DTP, Hib, Hepatitis B, IPV, PCV, Rotavirus, MMR, COVID-19, Flu vaccine, TCV, Japanese Encephalitis	Penta (DTwP/DPT + Hib + Hepatitis B), Polio (IPV), Rotavirus, MR, Japanese Encephalitis, PCV	DTaP, Hib, Hepatitis B, IPV, PCV, Rotavirus, MMR, COVID-19, Flu vaccine, TCV, Japanese Encephalitis
Child	Chickenpox vaccine, DTaP, Flu Vaccine, Hepatitis A, Hepatitis B, Hib, IPV, MMR, PCV, Japanese Encephalitis	DTwP/DPT, Polio (IPV), MR, Japanese Encephalitis.	Chickenpox vaccine, DTaP, Flu Vaccine, Hepatitis A, Hepatitis B, Hib, IPV, MMR, PCV, Flu Vaccine, Japanese Encephalitis.
Adolescent	Flu Vaccine, HPV Vaccine, MenACWY vaccine, Tdap, MenB***	Routine vaccines: Td, Japanese Encephalitis###. Non-routine vaccines: HPV, COVID-19.	Flu Vaccine, HPV Vaccine, MenACWY vaccine, Tdap, MenB***
Adult	Chickenpox, COVID-19, Flu Vaccine, Hepatitis B, HPV, MMR, Td or Tdap, Pneumococcal vaccine^^, Shingles vaccine^^, Mpox.	Non-routine vaccines: Japanese Encephalitis###, COVID-19.	Chickenpox, COVID-19, Flu Vaccine, Hepatitis B, HPV, MMR, Td or Tdap, Pneumococcal vaccine^^, Shingles vaccine^^, Mpox.
Older age group	COVID-19, Flu Vaccine, Pneumococcal vaccine, Shingles vaccine, RSV, Td or Tdap, Mpox	Non-routine vaccines: Japanese Encephalitis###, COVID-19.	COVID-19, Flu Vaccine, Pneumococcal vaccine, Shingles vaccine, RSV, Td or Tdap, Mpox.

*Recommended if pregnant person is at risk for a hepatitis A virus (HAV) infection or severe outcome from infection, if not previously vaccinated; **May be used if benefit outweighs risk; ***Base decision on risk vs. benefit; #High risk of exposure—may be used; ##If clearly needed; ^May be used if otherwise indicated; ^^Adults 50 years and older; ###The JE vaccination was started in 42 districts of three states where JE incidence is occurring among the adult population, namely Assam (9 districts), Uttar Pradesh (7 districts), and West Bengal (26 districts). JENVAC JE vaccine is used for the adult population (15+) in India.

The comparison between WHO-classified vaccines (Table 1), the global vaccination schedule (derived from the CDC schedule, 2023), and India’s immunization programs highlights critical areas with the potential to expand vaccine access (Tables 1, 2). While WHO-recommended vaccines address a broad spectrum of diseases, India’s national immunization schedule predominantly focuses on pediatric and select high-risk groups. Essential vaccines such as Diphtheria, Hepatitis B, *Haemophilus influenzae* type b (Hib), Measles, Pertussis, Polio, Rotavirus, Rubella, and Tetanus form the backbone of the UIP (32). However, significant gaps exist in adolescent and adult immunization coverage, particularly for vaccines like Hepatitis A, Influenza, Varicella, and Typhoid, which are largely restricted to the private sector. This creates inequities, as cost barriers may limit access for lower-income populations (33). Although the HPV vaccine largely remained limited to the private health sector, the government of India launched a National HPV Vaccination Campaign in 2026. Sustaining these gains will require a gradual transition toward routine delivery under the UIP to ensure long-term coverage and equitable access for eligible beneficiaries (34, 35). Japanese Encephalitis (JE) campaigns are limited to endemic states, focusing primarily on children, and often exclude broader adult and older adults populations due to limited surveillance data on disease burden in these groups (36). Global surveillance of VPDs is robust for childhood diseases but remains underdeveloped for adult and older adults populations, making it challenging to assess disease burden and prioritize vaccine introduction (37). Expanding the reach of these vaccines within the national immunization schedule, alongside enhancing real-time surveillance systems for emerging diseases across broader age groups, particularly adolescents, adults, and the older adults, could significantly reduce the burden of VPDs and align India more closely with global immunization strategies.

## 3 Critical factors for implementing a life course approach

### 3.1 LCI feasibility matrix

One of the most essential aspects of implementing a life course approach to immunization programs is assessing the factors countries should consider. These decisions are complex and multifactorial, varying by age cohort and disease burden, and the National Immunization Technical Advisory Groups (NITAGs) are responsible for making these recommendations. Some key factors that have emerged from the literature —disease burden and epidemiology, potential health impact, quality of life considerations, economic value and financing mechanisms, health system components like delivery platforms, competing health priorities, country’s capacity, multisectoral collaboration, and political will (37). These factors have been categorized into two major themes—"Population and Epidemiology” and “Health Systems and Program Delivery.” Figure 3 illustrates these two themes, their components or key factors, and the interrelationships to guide countries. These factors are critical, dynamic, and interrelated and emerged from the existing literature.

**Figure 3:**
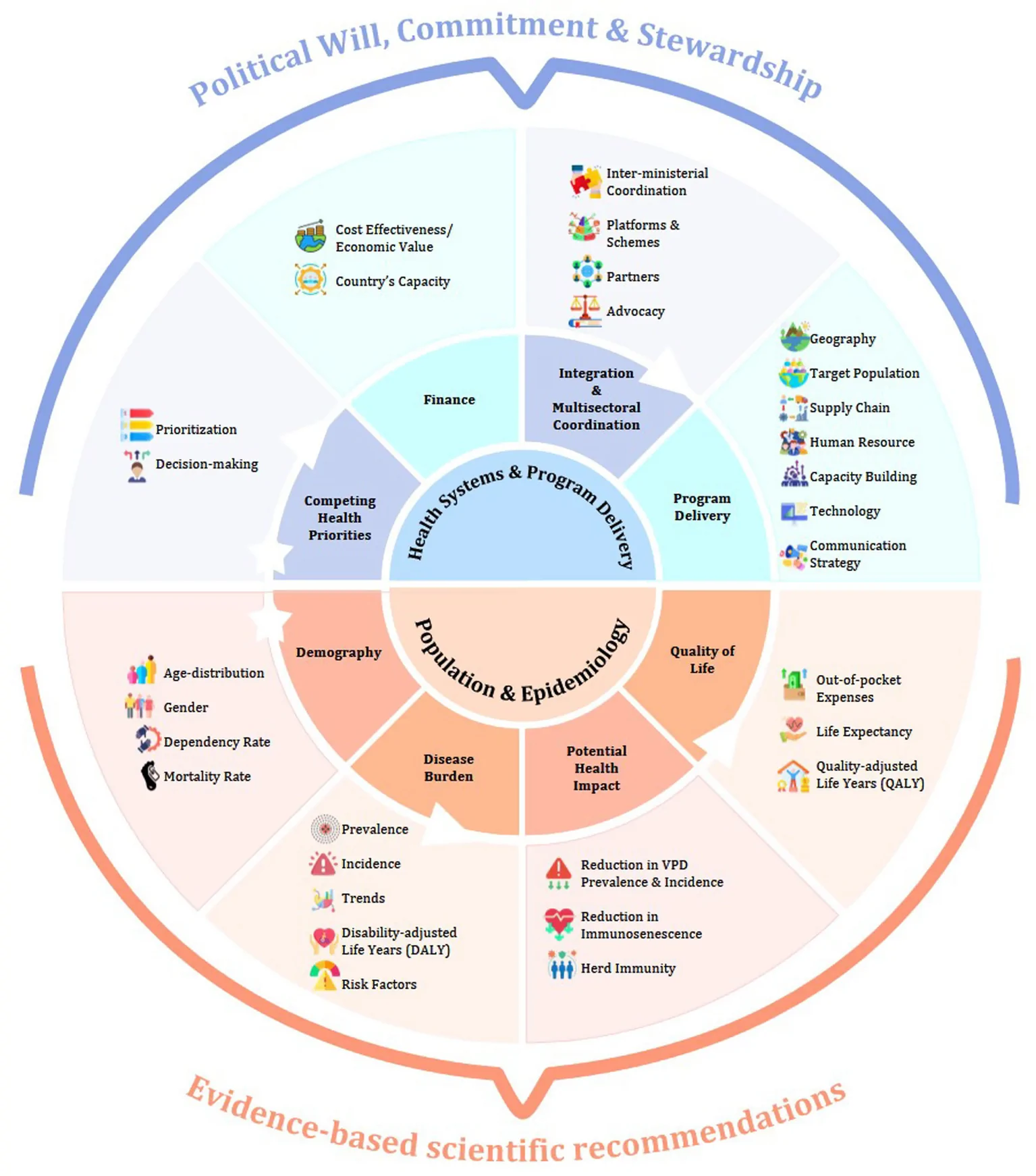
LCI factors feasibility matrix. This representation enumerates key factors to consider while planning and implementing LCI. These factors have been categorized into two major themes—‘Population and Epidemiology’ and ‘Health Systems and Program Delivery.’ Each theme encompasses four detailed factors. The matrix draws information from global examples and practices with the potential to inform LCA policies and planning in LMICs with resource constraints. This framework will be a foundational resource for scientific committees to advocate LCI and aid researchers in examining these factors, enabling policymakers to make informed and timely decisions.

#### 3.1.1 Population and epidemiology

This theme primarily focuses on establishing a need for a particular VPD and/or vaccine across the life course and the potential impact in a specific country. Investigating population characteristics and epidemiologic considerations is the first step in life course immunization planning. The national immunization working groups and experts derive scientific information from these factors, which is then evaluated, and *evidence-based scientific recommendations* are made.

#### 3.1.2 Demography

World Population Prospects 2019 has predicted a shift in the distribution of the global population toward older ages (4–6). This shift, which differs across regions and countries, is inclined toward younger populations in the African region and older populations aged 65 years and over in Southeast Asian countries, calling for the inclusion of adults and the older adults in national immunization programs. A high dependency ratio indicates that the economy and the working-age population (15–64 years) bear a greater burden to support the needs of dependent populations (0–14 and 65 + years), making it an essential factor in decision-making. High mortality rates can be examined, and mapping of VPDs that are responsible for these deaths can be done. Gender considerations are vital for assessing whether a disease is more prevalent in a particular gender, and important for developing gender-informed strategies and planning program delivery. Changing demographics are associated with changes in risk factors and disease susceptibility at different ages, and policymakers should recognize the need to ensure the recommended vaccines to maximize protection across the life course.

#### 3.1.3 Disease burden

Fundamentally, vaccines are administered before the onset of disease, so it is essential to generate evidence supporting age-appropriate vaccines across the life course, which will differ considerably in HICs and LMICs (37). Age is a deciding factor —age-related immunosenescence, associated risk factors, and vaccine efficacy are vital in determining adolescent and adult vaccination strategies. The vaccines should ideally be administered when a particular age cohort is the most susceptible to the specific disease or when the vaccine can most effectively prevent disease at a later stage (25). A VPD with high prevalence and incidence for which a WHO-approved vaccine is available and has been used in similar countries is a likely parameter to inform decision-making. Certain diseases are more prevalent during certain seasons or exacerbated by specific risk factors, such as diarrhea during monsoons and food-borne diseases like Typhoid, which need to be prioritized based on the country’s disease burden. Moreover, a different approach is required when a new vaccine is introduced in a particular cohort than when a booster dose is added to adult cohorts—with different implementing strategies, logistics, challenges, barriers to tackling vaccine hesitancy, etc. A lack of comprehensive epidemiological data on VPD burden among adults has been a limiting factor for LMICs in developing adult vaccination policies (38, 39). The Global Burden of Disease 2021 Forecasting for 2022–2050 states an expected shift—a decline in poor health outcomes and early deaths from Communicable, Maternal, Neonatal, and Nutritional (CMNN) and a rise in the burden of NCDs (40). This burden is measured in DALYs and is most likely to increase among adults and the older adults, and establishes a need to examine current and future disease trends. While vaccines primarily prevent infectious diseases, they also reduce the burden of chronic NCDs in adults and the older adults (41).

#### 3.1.4 Potential health impact

The long-term health benefits of vaccines in fighting pathogens, reducing disease burden, and ensuring healthy lives have been well established. A modeling study on EPI’s 50 years of impact estimates that 154 million deaths were averted from 1974 to 2024, with 95% of these occurring in children younger than five years (42). Protection from measles has been the highest contributor (43), and Measles and Rubella elimination is also the current regional and global priority. Reducing the VPD incidence and prevalence has a direct impact, and in the long run, immunization programs lead to “*herd immunity*,” which protects larger populations.

#### 3.1.5 Quality of life

Quality of life (QoL) is a multidimensional factor, and vaccination has been proven to improve QoL by increasing life expectancy and considerably reducing out-of-pocket (OOP) expenses. The quality-of-life factor has a greater impact on the older adults, who often face comorbidities and age-related health conditions. Reducing OOP expenses on VPDs across the life course allows people to invest in other health and non-health priorities and to reduce hospitalization costs. Immunization services contribute to 13 of the 17 Sustainable Development Goals (SDGs) directly or indirectly. It minimizes the impact of malnutrition, complements WASH, reduces disease transmission, increases educational attainment, ensures gender equality, mitigates the effects of climate change, and reduces inequalities by improving the lives of the most marginalized groups.

#### 3.1.6 Health systems and program delivery

Once countries are in a position to decide on a particular vaccine across the life course, for a specific age group and gender, informed by evidence-based scientific recommendations, the next step is to strategize implementation. The implementation, however, is complex and will depend on several factors grouped under the Health Systems and Program Delivery theme (Figure 3). This theme is largely guided and informed by political will, commitment, and stewardship at the national level.

#### 3.1.7 Competing health priorities

This factor calls for assessing the country’s situation and prioritizing vaccines and cohorts based on all the factors listed under the “Population and Epidemiology” theme—primarily the current disease burden, trends, and potential impact. This factor is of utmost importance in LMICs, countries with larger populations, and those with resource constraints. Given these limitations, LMICs often cannot include all the WHO-recommended vaccines in their national immunization programs simultaneously. Prioritization is crucial and will further inform the financial decisions based on the country’s budgetary strategies, GDP, and economic conditions.

#### 3.1.8 Finance

Financing is a critical component of LCA, as sustained and adequate funding is essential to ensure comprehensive vaccine coverage. A cost-effectiveness analysis, generating economic evidence to support life course interventions, is central to decision-making. These studies are particularly essential in countries with larger populations for estimating procurement and operational costs associated with introducing vaccines and integrating them into the national immunization program. It is even more crucial when countries introduce new vaccines and engage new cohorts. Financing mechanisms also need to be adapted to support LCI integration. This may involve pooling resources from different sectors, such as education, labor, and social welfare, to ensure that vaccination is adequately funded across the lifespan. Additionally, health insurance schemes and public health financing should cover vaccines for all age groups, not just children, to promote equitable access and encourage people to stay up to date with their vaccinations. Countries with well-established health insurance schemes or universal health coverage have more successfully integrated LCA into their policies. Secure funding channels ensure that vaccines are available, the health workforce is adequate and well-trained, and public awareness campaigns are effectively conducted.

#### 3.1.9 Integration and multi-sectoral coordination

LCA requires a robust multi-sectoral approach that involves collaboration across different ministries. Some key ministries that can be looped into the planning are women and child development, education, labor, and employment, etc.—inter-ministerial coordination with the Ministry of Health as an anchor, and these would differ across countries and with the target age cohort. This collaborative, integrated effort extends the reach of immunization programs beyond the health sector, addressing social determinants of health that affect vaccine uptake. Stakeholder engagement is vital for promoting the importance of lifelong immunization and ensuring that these programs are supported at all administrative levels. The LCA will require civil society organizations and non-profits to support advocacy at all levels. Platform and scheme identification is equally important—it is crucial to integrate immunization activities with the country’s existing health and non-health platforms and schemes to ensure communities’ trust in the health systems, minimize visits, and encourage vaccine uptake.

#### 3.1.10 Program delivery

The ultimate implementation of the life course vaccine involves several factors that need to be considered before incorporating it into the national immunization program. The planning will depend on whether the vaccine will be rolled out nationally, in specific states/regions, or in phases. The recommended target population is another factor to be considered—it is relatively easier to introduce a vaccine or booster dose within the existing cohort; however, there are several anticipated challenges in introducing a new age cohort into the immunization program. At this planning stage, it is recommended that countries draw lessons from other similar countries that have already implemented these vaccines in the relevant cohort to adapt them to their needs. Ensuring an uninterrupted supply chain, adequate workforce hiring and capacity building, and developing robust, tailored communication strategies to better advocate and minimize vaccine refusal are vital factors. Effective utilization of existing technology for various immunization data is essential. This comprehensive framework and approach have the potential to guide countries by assessing all the parameters essential to planning the life course cohort and adapting to the country’s needs.

### 3.2 Health insurance and LCI

One of the most common concerns about adopting a life course approach to immunization is the cost. Adding new vaccines at different life stages beyond early childhood puts pressure on public health budgets. This raises a crucial question: How can countries, especially those with limited resources, manage and sustain these costs? India’s growing health insurance network could provide an additional avenue for financing vaccination across the life course. The government-sponsored Ayushman Bharat–PM-JAY insurance scheme currently focuses mainly on inpatient and hospitalization services. Its benefit package could potentially be expanded or redesigned to include selected preventive and outpatient services, such as vaccination for adults and older adults. At the same time, the private health insurance sector in India is increasingly offering preventive health benefits through some insurance products, including annual health check-ups, outpatient consultations and, in some cases, vaccination. These two insurance pathways therefore offer different opportunities: government-sponsored insurance could potentially be expanded to include preventive vaccination as part of its benefit package, while private insurance could provide coverage where such benefits are already included in individual policies. Both mechanisms could be explored further to support vaccination against VPDs, particularly those associated with substantial morbidity, hospitalization and healthcare costs.

If vaccines help prevent these illnesses, hospital admissions will decline, thereby reducing insurers’ reimbursement burden. Studies have highlighted that in high-income countries, adult vaccine coverage is much higher among those who are insured (44). Private Insurance providers in those contexts often offer co-payments, discounts, or even reduced premiums to people vaccinated against certain diseases. If India explores similar models, health insurance (public and private) could become an important lever to support LCI roll-out.

### 3.3 Gender and LCI

Ensuring gender transformative immunization programs is critical to sustaining the gains of LCI. This ensures immunization programs are equitable, accessible, and effective for all genders, particularly women and girls who often face barriers to healthcare due to socio-cultural norms and economic constraints. Cancer of the cervix is prevalent in females and can be prevented by the HPV vaccine. The HPV vaccine is recommended for adolescent girls as a primary target; however, boys and older females are secondary targets, which varies across countries (45). In many settings, gender-related barriers, such as cultural resistance to vaccinating girls, lack of female autonomy, restricted access to health services, and vaccine-associated myths, have impeded the widespread adoption of this vaccine. In response, some countries like Rwanda have successfully implemented school-based immunization programs that reach girls directly; this makes it easier to obtain parental consent and disseminate knowledge among parents. The country has achieved remarkable HPV vaccine coverage by integrating vaccination with education and addressing gender-specific barriers through community engagement and obtaining parental consent (46). Similarly, Tetanus-Diphtheria (Td) vaccines administered during adolescence often miss girls due to gender biases in health service delivery. India’s voluntary HPV vaccination campaign mandates parental/guardian consent before vaccinating girls at a health facility. Consent is submitted via U-WIN or on a physical form if offline. At the facility, the health workers explain the process, obtain a signed consent form, and upload it to U-WIN to ensure a digital record.

Gender-sensitive approaches are vital to address these challenges. This includes training health workers on gender-responsive care, designing health communication strategies that target both men and women, and ensuring that immunization campaigns cater to all genders. For instance, in some African and South Asian countries, male involvement has been encouraged in maternal and child health programs to challenge traditional gender roles that limit women’s access to health services (47). Moreover, collecting and analyzing gender-disaggregated data is essential to identify gaps and monitor progress. Countries like India and Ethiopia have started integrating such data into their immunization programs, allowing targeted interventions that address the unique gender gaps (48).

### 3.4 Anti-microbial resistance and LCI

The life course approach to immunization offers a powerful strategy to mitigate the growing threat of antimicrobial resistance (AMR)—a critical global health challenge. AMR disproportionately affects adults and the older adults, who are more vulnerable to infections due to age-related immune decline, multimorbidity, and increased exposure to healthcare environments (49). Vaccination reduces the burden of infections that necessitate antibiotic use, lowering the opportunity for pathogens to adapt and mutate. This approach highlights the importance of immunization across all life stages, particularly for vulnerable groups such as older adults who face a higher risk of infection and antibiotic misuse (50).

Pneumococcal and influenza vaccines are particularly critical for older adults. Pneumococcal vaccines, including polysaccharide and conjugate formulations, prevent pneumonia and invasive pneumococcal diseases, both leading causes of hospitalization and antibiotic prescriptions among the older adults. Reducing these infections curtails the need for antibiotics, limiting the emergence and spread of resistant bacterial strains. Similarly, influenza vaccination prevents complications such as secondary bacterial pneumonia, which often require antibiotic treatment, thereby directly contributing to AMR reduction.

In addition to bacterial and viral infections, vaccines like HPV and Tetanus-Diphtheria (Td) indirectly support AMR control by preventing infections that might lead to procedures or complications requiring antibiotics. The HPV vaccine helps reduce the risk of surgeries prone to postoperative infections, while the tetanus vaccine prevents wound-related bacterial infections that would otherwise necessitate antibiotic treatment (51, 52). To maximize the impact of immunization, adult and older adults-focused immunization should be prioritized in combating AMR.

### 3.5 One health and LCI

Emerging zoonotic diseases and climate change are increasingly recognized as critical threats to global health. Climate-sensitive diseases such as malaria, dengue, Japanese Encephalitis, and Zika are on the rise, driven by the expanding geographical range and breeding periods of vectors like mosquitoes due to rising global temperatures. These changes lead to disease emergence in previously unaffected or moderately affected regions, amplifying their burden. This intersection of climate change, zoonotic diseases, and human health highlights the urgent need for a coordinated and integrated approach to prevention and control, with vaccination as a cornerstone strategy—*One Health* approach (53). CDC emphasizes addressing animal and environmental health to safeguard human health, warning that neglecting these areas undermines efforts to combat zoonotic diseases (54).

Younger populations may benefit from vaccines against diseases like Japanese Encephalitis and dengue. Older adults and immunocompromised individuals are more vulnerable to pneumococcal infections, Zika, influenza, and severe complications (55–57). Regions expected to experience a rise in vector-borne diseases due to climate change must prioritize vaccine accessibility for the most vulnerable populations. For instance, expanding immunization programs to include emerging zoonotic diseases in areas with increasing vector prevalence is critical. Vaccination programs for diseases such as malaria and Japanese encephalitis, already in place, can be scaled to include additional zoonotic diseases, thereby strengthening health systems.

### 3.6 Climate-resilient immunization program

Climate change poses a growing threat through the accelerated spread of climate-sensitive diseases, including malaria, dengue, Japanese Encephalitis, and Zika. Rising temperatures extend the breeding seasons of vectors like mosquitoes and expand their geographical range into temperate zones and previously unaffected regions. These shifts disproportionately impact vulnerable populations, including children, the older adults, and those with weakened immune systems (58). Climate change also introduces an emerging risk to vaccine efficacy, as shifts in temperature and humidity threaten the stability of vaccine supply chains. Strengthening the cold chain infrastructure, incorporating renewable energy solutions such as solar-powered cold storage, and investing in climate-resilient vaccine management systems will be essential to safeguard vaccine quality. These collectively call for building climate-resilient immunization programs that adapt to evolving epidemiological patterns by strengthening vaccine supply chains, enhancing disease surveillance, and fostering the development of vaccines for emerging threats.

Recent advancements in vaccines for Zika and dengue demonstrate the potential for innovative solutions to mitigate the impacts of climate-driven health risks (59). Equitable vaccine access is integral to this approach and can be ensured through improved microplanning, including the use of digitally enhanced, technology-aided microplans. Addressing social determinants of health, such as poverty, education, and healthcare access, is critical to improving vaccine uptake. Public health campaigns must also raise awareness about the role of vaccinations in climate resilience, encouraging individuals to maintain up-to-date immunizations as part of a broader strategy for disease prevention and healthy ageing.

## 4 Discussion

Integrating the LCI into existing health systems requires a holistic strategy that emphasizes multi-sectoral coordination and system-wide adaptability to address the evolving health needs of individuals across all stages of life. This requires a strong political commitment to positioning LCA as a part of the national-level strategy. National health authorities and health ministries must clearly articulate LCA’s benefits in health policies and align its goals with the broader SDGs. NITAGs have been pivotal in shaping immunization programs and introducing vaccines for various age groups. As of 2021, more than 80% of countries have established NITAGs, and 69% (119 of 172) meet the six process indicators of WHO to be categorized as functional; IA 2030 has also emphasized the need to strengthen their influence (60, 61). These expert groups provide essential guidance for integrating LCA into immunization programs, helping to prioritize vaccines and develop age-appropriate schedules. Life course immunization emerged as an important focus area and opportunity in the 15th and 18th NITAG-India, held in 2020 and 2023, respectively (62, 63). The absence of age-specific data on VPDs in the Indian population limits efforts to assess and address disease burdens across life stages accurately. While pediatric immunization is adequately covered, adult and older adults cohorts remain underrepresented. This gap disproportionately affects immunocompromised populations, heightening their vulnerability to infections. Without surveillance and resource allocation, adult immunization programs remain fragmented. Strengthening data collection is essential to reduce VPDs and ensure data-driven decision-making for lifecourse immunization.

Comprehensive Primary Healthcare (CPHC), an approach to achieving universal health coverage, can be leveraged to expand immunization services. This can be achieved by integrating vaccines into the CPHC framework by aligning vaccination schedules with other routine health services such as maternal and child health visits, school health programs, and geriatric care for older adults. This integration promotes a seamless transition between different life stages, ensuring that vaccination remains a continuous priority throughout a person’s life. One way to achieve this integration is to leverage existing systems, such as electronic health records (EHRs) and immunization registries, to track vaccination status throughout a person’s lifetime. These systems can be linked with other health services, such as screenings and chronic disease management, to ensure that vaccination is part of a broader preventive health strategy. For instance, when an adult visits a clinic for a routine health check-up, their vaccination status can be reviewed, and any missing vaccines recommended for adults can be administered with consent, reinforcing the importance of immunization at every stage of life. Vaccination coverage in the second year of life is often lower than in the first year. Implementation challenges are observed in the second year of life owing to the sub-optimal orientation of the frontline workers to new targets and delivery modes–this also applies to adolescent vaccination (64). In 2024, India launched UWIN, a name-based digital registry system to enhance the immunization program through efficient tracking and real-time monitoring. The platform also offers a self-registration feature for all eligible citizens. UWIN is inspired by the success of COWIN, a platform that boosted vaccination rates among adults and the older adults during the COVID-19 pandemic. This technological integration will pave the way for future lifecourse vaccines in the country (65, 66).

Multi-sectoral stakeholder engagement requires attention owing to the emerging complexities of health systems. Schools, workplaces, and community organizations can be vital in promoting and facilitating vaccination, especially for adolescents and adults. This approach enhances acceptability, ensures accessibility, reduces barriers, and improves coverage rates. School-based vaccination programs can be expanded to include vaccines for older children and adolescents, ensuring that this age group is protected against VPDs. LCI integration requires ongoing education and awareness campaigns to shift the public’s perception of vaccination from a childhood-only activity to a lifelong health behavior. Healthcare providers must be trained to deliver age-appropriate vaccination messages and to address vaccine hesitancy in all age groups. Public health campaigns should emphasize the benefits of a life course approach as new vaccines are introduced for the ageing population. To do so, countries need to allocate adequate resources to procure vaccines, regularly train healthcare workers, enhance infrastructure, and run public awareness campaigns. Incorporating expanded immunization services into health insurance schemes ensures equitable vaccine access, particularly for marginalized and vulnerable populations. Academic institutions and research bodies are indispensable for providing evidence-based guidance and evaluating the effectiveness of immunization programs. Strong partnerships with the private sector, particularly with pharmaceutical companies and technology firms, ensure the availability, affordability, and effective monitoring of vaccines through data-driven platforms. Public engagement is another critical component. Effective communication strategies must address vaccine hesitancy and misinformation, particularly among adults and the older adults. Involving community leaders and influencers can help build trust and acceptance, particularly when introducing vaccines for new age groups. Robust monitoring and evaluation mechanisms, tracking vaccine coverage and disease prevalence, are essential. These systems should track vaccine coverage, disease incidence, and health outcomes across age groups, providing data to identify gaps and refine strategies, and creating demand. Regular assessments ensure that immunization programs remain responsive to the population’s changing needs. Aligning national policies with global frameworks, such as IA 2030 and WHO’s strategies for healthy ageing, enables countries to benefit from shared resources and technical support while advancing global health goals.

Systemic barriers and perceptions exist amongst adult and older adults cohorts. Immunization coverage among adults and the older adults faces unique challenges shaped by biological, social, and systemic factors. As age-associated vulnerability increases, vaccines are critical for these populations. Gaps in vaccination programs, limited awareness, and logistical barriers often hinder access and acceptance of vaccines in these cohorts. These challenges are compounded by the underrepresentation of adult and older adults immunization in national health strategies. Among the barriers to adult and older adults immunization, vaccine hesitancy and resistance remain significant obstacles. These issues are often rooted in longstanding beliefs, perceptions, and mistrust in the healthcare systems. Many adults and older individuals perceive vaccines as primary preventive tools for children, leading to a diminished sense of urgency about immunization in later life. This misconception is particularly evident with vaccines such as those for influenza, pneumococcal disease, and shingles, which are vital for protecting against severe illnesses but remain underutilized (67).

Misconceptions about vaccine safety and efficacy can persist and intensify without clear, accessible information. Public health campaigns should highlight the role of vaccines in reducing hospitalizations, preventing complications, and supporting healthy ageing. Community leaders, faith-based influencers, and civil society groups must be mobilized to build trust, combat vaccine hesitancy, and address social and cultural barriers. NGOs and patient advocacy groups play a crucial role in outreach, advocacy, and education, helping to ensure that all segments of the population, especially the vulnerable, are reached (68). Fear of adverse events following immunization (AEFIs) also poses a significant barrier to vaccine acceptance among older populations. Even mild or moderate AEFIs can deter future vaccination, particularly for individuals with heightened health concerns. Addressing these fears requires transparent communication about the risks and benefits of vaccines. Developing robust monitoring systems for AEFIs and equipping healthcare providers with the tools to manage and reassure patients are critical to building trust and confidence in immunization (69).

The development and delivery of vaccines for adults and the older adults must also account for age-related changes in immune function. Formulations that are both effective and well-tolerated are essential to ensure uptake. However, the success of these vaccines ultimately depends on demand generation through targeted education and outreach efforts. Healthcare providers play a pivotal role in this process, engaging in meaningful conversations with beneficiaries, addressing their concerns, and reinforcing the importance of immunization in preventing severe illness and maintaining quality of life. By addressing these interconnected challenges—systemic gaps, hesitancy, misinformation, and fear of AEFIs—immunization programs can better serve adult and older adults populations.

The COVID-19 pandemic has provided critical insights into the importance of LCI. The rapid development and global rollout of COVID-19 vaccines highlighted the need for vaccination strategies that extend beyond childhood and target all age groups (70). The pandemic highlighted the vulnerabilities of older adults and those with underlying health conditions, who faced higher risks of severe illness and death from COVID-19. This situation demonstrated the need for integrated adult and older adults vaccination programs within routine healthcare services. The success of COVID-19 vaccination efforts in reducing hospitalizations and mortality among these populations emphasizes the value of an LCA in ensuring continuous protection against VPDs (71, 72).

Significant challenges related to vaccine hesitancy, particularly among adults and the older adults, emerged strongly. The spread of misinformation, coupled with pre-existing mistrust in vaccines, led to varying levels of vaccine acceptance across different demographic groups. The COVID-19 pandemic demonstrated that effective communication strategies and community engagement are essential for overcoming hesitancy and ensuring high vaccine uptake across the life course. Vaccine inequity and distribution emerged as another critical lesson. The disparities observed in vaccine access and uptake highlighted the need for policies that ensure equitable access for all age groups, particularly marginalized populations (73). Logistical challenges, such as the need for cold chain infrastructure and extensive coordination, emerged during the COVID-19 vaccination rollout. These challenges highlight the need for health systems to be resilient and adaptable and respond adequately to public health emergencies. Building such capacity is essential for successfully implementing LCI. The pandemic also reinforced the importance of global collaboration in vaccine development and distribution. International cooperation and shared resources enabled the unprecedented speed at which COVID-19 vaccines were developed, tested, and distributed. This collaborative approach is equally critical for the successful implementation of LCI, as it requires ongoing research, development, and the sharing of best practices to address the evolving landscape of VPDs.

India’s immunization efforts have made significant progress in providing vaccines for twelve preventable diseases (74). However, this program has primarily focused on childhood vaccination and immunization for pregnant women, leaving considerable gaps in coverage across the life course. The comprehensive integration of LCI presents an opportunity to strengthen further efforts in addressing current and emerging public health challenges (12). Some state-level initiatives have expanded immunization to include adolescents and adults in recent years. Healthcare workers are prioritized for Hepatitis B vaccination due to their occupational risks. Japanese encephalitis vaccines are administered to adults in endemic states of the country, and tetanus and diphtheria (Td) vaccines are routinely given to pregnant women to safeguard maternal and neonatal health under UIP (75). Many vaccines for adults, such as Hepatitis B, typhoid, influenza, pneumococcal, and shingles, are available through the private healthcare sector, where their high cost limits uptake, leading to disparities. To accelerate the elimination of tuberculosis, the Government of India launched the “Adult BCG Vaccination Study” in 2024 under the National Tuberculosis Elimination Program (NTEP), aligning with tuberculosis eradication (76). The study aims to assess the effectiveness of the BCG vaccine in adults, expanding its use beyond childhood immunization, where it is already part of the UIP. While this represents significant progress, developing comprehensive national guidelines for adult vaccination is crucial. Establishing such frameworks would promote a more inclusive immunization strategy.

The growing burden of VPDs among adolescents and adults in several LMICs, including India, underlines the need for a more comprehensive immunization strategy. There has been an increase in typhoid cases in recent years due to several factors, such as increased urbanization, AMR, and the impact of climate change (77). These highlight an opportunity to strengthen immunization efforts by adding new vaccines and booster doses, particularly among adolescents and adults. Vaccines like pneumococcal and shingles, recommended for older adults in many countries, are not yet systematically included in India’s public health programs despite their clear benefits in preventing hospitalizations and deaths.

Adolescence is a critical life stage for immunization, as immunity against certain diseases wanes during this period, requiring booster doses to sustain protection. During adolescence, booster doses of tetanus, diphtheria, and pertussis (Tdap) vaccines can help maintain immunity and protect against these diseases. The HPV vaccine, which has demonstrated efficacy in preventing cervical cancer, is administered to adolescent girls through a nationwide campaign. Maternal immunization also plays a vital role in protecting both mothers and newborns. Yet, opportunities exist to expand coverage beyond tetanus to include other vaccines, such as influenza, which can provide additional protection against respiratory infections. The absence of a robust adult vaccination program results in inadequate protection against VPDs for adults and older populations, further compounded by high vaccine costs and limited awareness of their benefits. To address these gaps, the NTAGI has recommended incorporating the typhoid conjugate vaccine (TCV) into the routine immunization program, improving affordability and coverage, and reducing disease burden (78). Expanding the scope of LCI in India would also include critical vaccines like pneumococcal, shingles, and influenza, which could significantly reduce morbidity and healthcare costs (79). However, countries like India may not need to introduce all WHO-recommended vaccines. The country would rather benefit more from disease burden and surveillance-informed data across age cohorts and competing priorities. The economic benefits of vaccination extend beyond individual health to include reductions in healthcare expenditures and lower burdens on public health infrastructure, particularly during disease outbreaks. Implementing LCI in India requires comprehensive systemic reforms, including expanding the existing immunization framework to cover all age groups, introducing new vaccines, and establishing platform-based regulatory models to enable the swift introduction and scale-up of vaccines.

India stands at a critical juncture in its public health landscape, where adopting LCI policies can be transformative in addressing the rising burden of VPDs. India has built a strong foundation that can now be expanded to encompass all age groups. The COVID-19 pandemic has reinforced the need for integrated life course vaccination policies to respond to emerging health threats (80). India’s preparedness is underpinned by its existing strengths. It is the largest vaccine manufacturer in the world, with extensive production capabilities and an expanding network of healthcare delivery points from primary to tertiary levels. India is known for its proficiency in manufacturing cost-effective vaccines and executing large-scale operations using the most advanced technology (81, 82). The country can achieve self-sufficiency in meeting its increasing demand for lifecourse vaccines. Additionally, the government’s commitment to immunization programs is evident in initiatives such as the Intensified Mission Indradhanush, a large-scale catch-up campaign. These robust systems and campaigns allow the government to vaccinate leftout and zerodose children nationwide–a step essential to the elimination of diseases like Measles and Rubella. NTAGI has consistently guided and evolved the country’s immunization policies. Over the last 5 years, NTAGI and other expert working groups have highlighted the need to expand beyond childhood vaccination. Leveraging NTAGI’s existing frameworks, incorporating recent evidence, and integrating cross-sectoral recommendations can help develop a cohesive, phased LCI strategy. By addressing gaps in adolescent and adult vaccination coverage, India can reduce the incidence of diseases such as cervical cancer, Hepatitis B, typhoid, tuberculosis, and pneumococcal infections, which disproportionately affect these populations (83, 84).

India’s existing health platforms offer a strong foundation to integrate immunization into routine healthcare to reach people across the life stages. Leveraging each platform’s unique target population, contacts, and workforce can establish a continuous life-course care package. For example, the National Program for Health Care of the Elderly (NPHCE), launched in 2010–11, delivers care across health and wellness centers, primary and community health centers, district hospitals, Regional Geriatric Centers, and home visits. Its structured referral pathway across primary, secondary, and tertiary care can be an opportunity to review vaccination status during geriatric consultations and recommend or offer eligible vaccines, embedding immunization within healthy ageing services.

The Rashtriya Kishor Swasthya Karyakram (RKSK), launched in 2014, offers a distinct platform for adolescent health. Operating across various facility tiers, community settings, and schools, it provides an age-responsive entry point beyond traditional maternal and child health services to link adolescent immunization. Similarly, schools reach large numbers of youth, providing another high-value platform. Through the Ayushman Bharat School Health and Wellness Program, trained teachers designated as Health and Wellness Ambassadors deliver health promotion via health-education convergence. This platform can facilitate age-based immunization through scheduled sessions, parental communication, identification of missed doses, and referral of students to health facilities requiring vaccination.

However, the presence of delivery platforms does not mean that expanding immunization across the life course would be resource-neutral. Allocating additional public resources creates opportunity costs, including health budgets, health worker time, cold chain, storage and logistics, training, and reporting. Feasibility depends not just on system integration, but on what other health priorities lose resources as a result. Importantly, India-specific economic evaluations are already available for some vaccines, demonstrating how evidence can inform such prioritization. A cost-effectiveness analysis of TCV in India showed cost savings in urban settings but not in rural areas due to differences in disease incidence and costs (85). Similarly, an economic evaluation of HPV vaccination in India found that vaccination of adolescent girls was cost-effective, although the results were sensitive to vaccine price and delivery costs. The study estimated a total vaccine and delivery cost of US$24.2 per fully immunized girl (86).

At the same time, India’s broader health-financing context is also critical to assessing the feasibility of a life-course approach. National Health Accounts estimates show that public health expenditure in India has increased over time, with combined central and state spending reaching ~1.8–1.9% of GDP. The Government of India has also set a target to increase public health expenditure to 2.5% of GDP (87). Additionally, health is primarily a state subject, with the Union Government extending financial and technical support via national health programs. Rethinking immunization programs through a life course perspective offers a transformative approach to addressing evolving VPDs. This approach is particularly critical in light of global demographic shifts, including increased longevity, intensifying the health needs of ageing populations and persistent social inequalities.

Immunosenescence in older adults and immunity gaps in other age groups amplify vulnerabilities to diseases like influenza, pneumococcal infections, and shingles. Expanding national immunization programs offers a path to comprehensively address these vulnerabilities. India’s immunization journey highlights significant achievements in childhood vaccination, but it also underscores the pressing need to strengthen and expand policies and services for adolescents and adults. Integrating LCA into India’s UIP would strengthen its ability to address VPDs and align with broader public health goals. By adopting LCA, India can address challenges such as zoonotic diseases, climate change-related health risks, and emerging public health emergencies, ensuring a resilient health system. Globally, LCA has demonstrated its potential to reduce disease burden and improve population-level health outcomes. Integrating the life course approach into national and global immunization programs is no longer an option but a necessity.

## 5 Conclusion

India’s changing demographic and disease profile provides a strong rationale for moving toward a life-course approach to immunization, while recognizing that childhood immunization must remain a core priority. India already has health-system platforms that can reach adolescents, adults, and older adults through routine health and community services. However, expansion should be gradual and guided by evidence on disease burden, vaccine effectiveness, cost-effectiveness, equity, affordability and implementation feasibility. India-specific economic evaluations and existing health programs can help identify which vaccines and population groups to prioritize. A well-planned approach can strengthen preventive health across the life course without creating parallel systems or placing avoidable pressure on frontline health workers. The next step is to generate stronger India-specific evidence and develop a clear, prioritized and sustainable framework for life-course immunization.

### 5.1 Limitation

This review has some limitations that should be considered when interpreting and correlating the findings. First, although the manuscript discusses the potential role of immunization across the life course, it does not undertake a formal economic evaluation or costing of an adult vaccination program in India. The financial implications would depend on the vaccines considered, target population, coverage, vaccine prices, delivery platforms, human-resource requirements, and supply-chain arrangements, and these parameters may vary considerably across different implementation models. Similarly, the review does not quantify the additional workload a life-course approach may place on frontline health workers, as this depends on how and at what level adult vaccination is integrated into the existing health system. Second, the strength of evidence is not uniform across all adult vaccines and population groups; therefore, the narrative should not be interpreted as implying that all adult vaccines have the same level of evidence, effectiveness, cost-effectiveness, or priority for inclusion in the national program. Future research should include India-specific costing, workforce and implementation assessments, and vaccine-specific evaluations to better understand the resource implications, feasibility, and potential opportunity costs of expanding immunization across the life course.
