# Supplementary material for: Lifecourse immunization and adaptation to changing needs of India’s universal immunization program: theory to practice
Source: Front Public Health. 2026 Oct 28;14:1938980. doi: 10.3389/fpubh.2026.1938980 (PMC13752830; doi:10.3389/fpubh.2026.1938980)
Supplement: Supplementary file 1 [file Table_1.docx]

# Supplementary Table 1: Reported cases of vaccine-preventable diseases (VPDs), India and global, 2000–2025

| Country | Disease | 2025 | 2024 | 2023 | 2022 | 2021 | 2020 | 2019 | 2018 | 2017 | 2016 | 2015 | 2014 | 2013 | 2012 | 2011 | 2010 | 2009 | 2008 | 2007 | 2006 | 2005 | 2004 | 2003 | 2002 | 2001 | 2000 |
| --- | --- | --- | --- | --- | --- | --- | --- | --- | --- | --- | --- | --- | --- | --- | --- | --- | --- | --- | --- | --- | --- | --- | --- | --- | --- | --- | --- |
| **India** | Diphtheria | 3,249 | 5,634 | 3,850 | 3,286 | 1,768 | 3,485 | 9,622 | 8,788 | 5,293 | 3,380 | 2,365 | 6,094 | 3,133 | 2,525 | 4,233 | 3,434 | 3,529 | 3,977 | 3,812 | 2,834 | 5,826 | 8,465 | 4,236 | 5,301 | 5,472 | 5,125 |
| **Global** | Diphtheria | 30,205 | 25,147 | 24,782 | 10,027 | 8,659 | 10,137 | 22,989 | 16,911 | 8,819 | 7,102 | 4,535 | 7,774 | 4,680 | 4,490 | 5,626 | 4,603 | 4,349 | 4,978 | 4,642 | 4,333 | 8,137 | 10,069 | 7,154 | 9,035 | 10,725 | 11,625 |
| **India** | Congenital rubella syndrome | 72 |  | 63 | 210 |  |  |  |  | 76 | 25 |  |  |  |  |  |  |  |  |  |  |  |  |  |  |  |  |
| **Global** | Congenital rubella syndrome | 746 | 611 | 1,534 | 1,527 | 716 | 1,252 | 424 | 449 | 835 | 369 | 282 | 142 | 134 | 302 | 214 | 31 | 161 | 243 | 225 | 64 | 41 | 89 | 116 | 41 | 85 | 156 |
| **India** | Invasive meningococcal disease | 77 |  |  |  |  |  |  |  |  |  |  |  |  |  |  |  |  |  |  |  |  |  |  |  |  |  |
| **Global** | Invasive meningococcal disease | 20,680 | 14,926 | 5,985 | 16,323 | 26,059 | 21,126 |  |  |  |  |  |  |  |  |  |  |  |  |  |  |  |  |  |  |  |  |
| **Global** | Japanese encephalitis | 2,110 | 576 | 2,120 | 1,886 | 988 | 1,532 | 3,709 | 4,419 | 4,668 | 5,427 | 4,086 | 4,810 | 3,871 | 2,351 | 3,392 | 3,856 | 5,163 | 5,198 | 9,487 | 8,040 | 84 | 39 | 38 | 55 | 1 | 0 |
| **India** | Japanese encephalitis | 1,414 |  | 1,320 | 1,271 | 489 | 718 | 2,496 | 1,707 | 2,043 | 1,627 | 1,620 | 1,657 | 1,078 |  | 1,214 | 555 | 653 | 427 | 4,017 |  |  |  |  |  |  |  |
| **India** | Measles | 17,995 | 18,530 | 65,150 | 40,967 | 5,700 | 5,604 | 10,430 | 19,474 | 12,032 | 17,250 | 30,168 | 26,530 | 8,285 | 3,305 | 33,634 | 31,458 | 56,188 | 44,258 | 41,144 | 64,185 | 36,711 | 55,443 | 47,147 | 40,044 | 51,780 | 38,835 |
| **Global** | Measles | 4,11,913 | 4,76,412 | 6,69,083 | 2,06,775 | 1,23,152 | 1,59,240 | 8,74,099 | 3,59,295 | 1,73,457 | 1,32,490 | 2,14,808 | 2,82,078 | 2,75,307 | 2,12,376 | 3,59,332 | 3,43,806 | 2,78,637 | 2,78,751 | 2,85,031 | 3,77,576 | 5,85,701 | 5,13,406 | 6,79,856 | 5,86,471 | 8,49,173 | 8,53,479 |
| **Global** | Mumps | 2,35,043 | 2,45,062 | 3,87,586 | 3,81,029 | 2,41,742 | 2,79,270 | 1,69,898 | 5,02,132 | 5,60,784 | 5,92,174 | 3,85,781 | 3,11,602 | 5,16,316 | 6,87,934 | 7,26,638 | 6,19,389 | 5,46,684 | 5,37,740 | 4,07,873 | 6,43,321 | 6,19,062 | 6,54,216 | 3,34,524 | 4,87,932 | 4,12,341 | 5,44,093 |
| **India** | Mumps | 339 |  | 195 | 6 | 758 |  |  |  |  |  |  |  |  |  |  |  |  |  |  |  |  |  |  |  |  |  |
| **Global** | Pertussis | 2,65,305 | 9,41,893 | 1,63,400 | 64,313 | 30,402 | 69,605 | 1,49,849 | 1,69,240 | 1,63,030 | 1,74,624 | 1,56,065 | 1,86,438 | 1,72,109 | 2,50,330 | 1,73,441 | 1,61,234 | 1,66,592 | 1,48,095 | 1,39,521 | 1,27,410 | 1,52,784 | 2,45,377 | 1,16,598 | 1,35,944 | 1,74,847 | 1,90,475 |
| **India** | Pertussis | 2,149 | 2,395 | 4,949 | 4,362 | 593 | 12,566 | 11,875 | 13,208 | 23,766 | 37,274 | 25,206 | 46,706 | 31,089 | 44,154 | 39,091 | 40,508 | 60,385 | 43,697 | 46,674 | 30,088 | 31,122 | 32,786 | 33,954 | 33,289 | 34,703 | 31,431 |
| **Global** | Poliomyelitis | 293 | 418 | 540 | 865 | 700 | 1,113 | 378 | 137 | 118 | 42 | 106 | 415 | 482 | 294 | 717 | 1,412 | 1,789 | 1,736 | 1,390 | 2,023 | 2,033 | 1,256 | 784 | 1,922 | 496 | 2,971 |
| **India** | Poliomyelitis |  |  |  |  | 0 | 0 | 0 | 0 | 0 | 0 | 0 | 0 | 0 | 0 | 1 | 44 | 756 | 559 | 874 | 676 | 66 | 134 | 225 | 1,600 | 268 | 265 |
| **Global** | Rubella | 27,840 | 31,244 | 35,731 | 18,881 | 10,010 | 10,317 | 49,180 | 26,033 | 14,756 | 23,419 | 23,760 | 33,516 | 1,01,156 | 94,277 | 1,14,456 | 75,961 | 1,21,378 | 1,81,029 | 1,96,501 | 2,50,102 | 2,67,983 | 3,08,213 | 3,17,415 | 6,39,747 | 8,36,332 | 6,70,894 |
| **India** | Rubella | 2,813 | 2,600 | 2,952 | 2,525 | 1,675 | 1,313 | 3,404 | 2,328 | 2,748 | 8,274 | 3,252 | 4,870 | 3,698 | 1,232 |  |  |  |  |  |  |  |  |  |  |  |  |
| **India** | Neonatal tetanus | 38 | 70 | 62 | 65 | 81 | 162 | 35 | 129 | 295 | 227 | 491 | 492 | 415 | 588 | 734 | 521 | 898 | 876 | 1,076 | 625 | 821 | 1,183 | 1,720 | 1,580 | 1,718 | 3,287 |
| **India** | Total tetanus | 38* | 70* | 62* | 65* | 1,240 | 1,361 | 7,071 | 7,000 | 4,946 | 3,781 | 2,268 | 5,017 | 2,814 | 2,404 | 2,843 | 1,756 | 2,126 | 2,959 | 7,491 | 2,815 | 2,981 | 3,883 | 4,020 | 12,197 | 5,764 | 8,997 |
| **Global** | Neonatal tetanus | 2,458 | 3,368 | 4,585 | 2,099 | 4,140 | 2,300 | 2,173 | 1,803 | 2,266 | 1,997 | 3,580 | 2,238 | 4,149 | 4,654 | 4,295 | 5,085 | 5,611 | 6,635 | 6,225 | 8,416 | 9,848 | 9,414 | 9,043 | 11,633 | 14,017 | 17,935 |
| **Global** | Total tetanus | 27,200 | 22,821 | 21,890 | 6,705 | 9,828 | 11,897 | 14,745 | 15,103 | 12,509 | 13,813 | 10,338 | 12,531 | 13,532 | 10,475 | 12,649 | 11,181 | 11,981 | 15,850 | 20,361 | 15,305 | 15,675 | 13,987 | 14,220 | 31,376 | 20,800 | 23,711 |
| **Global** | Typhoid | 42,82,076 | 1,33,95,970 | 45,83,555 | 28,97,145 | 18,58,715 | 8,88,757 | 1,48,557 | 7,456 | 34 | 35 | 32 | 31 | 19 |  |  |  |  |  |  |  |  |  |  |  |  |  |
| **India** | Typhoid | 5,68,088 | 6,09,018 | 5,74,129 | 4,02,532 |  |  |  |  |  |  |  |  |  |  |  |  |  |  |  |  |  |  |  |  |  |  |
| **India** | Yellow fever |  |  |  |  |  |  |  |  |  |  |  |  |  |  |  |  |  |  |  |  |  |  |  |  | 0 |  |
| **Global** | Yellow fever | 632 | 513 | 476 | 217 | 281 | 1,139 | 459 | 2,064 | 876 | 1,154 | 72 | 54 | 294 | 262 | 2,591 | 737 | 136 | 372 | 265 | 358 | 591 | 1,376 | 739 | 715 | 656 | 699 |

Source: WHO Immunization Data portal, reported cases of vaccine-preventable diseases, collected annually through the WHO/UNICEF Joint Reporting Form on Immunization (poliomyelitis: official country reporting to WHO); accessed 21 October 2026. WHO revises earlier years as countries update their reports. Global figures are the sum of WHO Member States’ reports and include India. Blank cells: no data reported. Total tetanus includes neonatal tetanus. *India did not report non-neonatal tetanus for 2022–2025, so its total tetanus figures for these years equal its neonatal counts. India did not report Japanese encephalitis, congenital rubella syndrome or mumps for 2024. Typhoid figures are not comparable across years: only Belgium reported typhoid before 2018, India began reporting in 2022, and the 2024 global figure includes 8,719,996 cases reported by Nigeria.
